# Development of sex-linked markers for gender identification of *Actinidia arguta*

**DOI:** 10.1038/s41598-023-39561-0

**Published:** 2023-08-07

**Authors:** Dandan Guo, Ran Wang, Jinbao Fang, Yunpeng Zhong, Xiujuan Qi

**Affiliations:** Key Laboratory for Fruit Tree Growth, Development and Quality Control, Zhengzhou Fruit Research Institute, Chinese Academy of Agricultural Sciences, Zhengzhou, 450009 China

**Keywords:** Molecular biology, Plant sciences

## Abstract

The fruit of the dioecious plant *Actinidia arguta* has become a great attraction recently. It has long been difficult to distinguish the genders of hybrid seedlings before flowering, therefore increasing the expenditures of breeding. To produce reliable molecular marker for gender identification, this research utilized whole-genome re-sequencing of 15 males and 15 females from an 8-year-old cross population to develop gender specific markers. P51 and P11 were identified as sex-linked markers after verification. Both of these markers, according to the PCR results, only amplified a single band in male samples. These two markers were tested in 97 hybrids (52 females and 45 males) and 31 wild individuals (13 females and 18 males), with an accuracy of 96.88% and 96.09%, correspondingly. This research also verified the universalities of the two markers in *Actinidia chinensis* samples, and it could be inferred from the PCR results that neither marker was applicable to *A. chinensis* samples. The BLAST results of the two markers demonstrated that the two markers were closely aligned with different parts of the Y male-specific region of *A. chinensis* genome, thus they were likely to be useful for the research on the mechanism of sex determination of *A. arguta*. The two male-linked makers, P51 and P11, have already been used in sex-identification of *A. arguta* seedlings.

## Introduction

The commercialization of kiwifruit began in the early twentieth century. Over the past 100 years, large fruit types, including cultivars of *Actinidia chinensis* and *Actinidia deliciosa*, have been dominating the market. However, the fruit of *Actinidia arguta* (kiwiberry) is receiving growing consumer acceptance around the world. The *A. arguta* fruit is small, hairless, and ready-to-eat, once the fruit is ripe. Ripe *A. arguta* fruits are green or red, extensively rich in medicinal ingredients and low in calories^1^. Besides, the full red type is also rich in anthocyanins^2^, which are now considered as an important kind of nutrients to the human body. The cultivation of *A. arguta* is a new addition to the kiwifruit industry, and it is developing rapidly in urban agriculture. The *A. arguta* plants are strongly resistant to cold stress and diseases^3,4^. The *A. arguta* species is widely distributed in China, ranging from tropical areas to cold temperate regions^5^. Thus, *A. arguta* offers an immense germplasm resource for the kiwifruit industry and for breeding practices.

Like other species of kiwifruit, the plants of *A. arguta* are functionally dioecious. The sex determination mechanism of kiwifruit plants is divided into XX/XY type^6–8^, and the sex-determination region of kiwifruit was located in the subtelomeric region of chromosome 25^9^, where tandem repeats with different lengths and repetitiveness levels exists^10^. There is no visual difference between the female and male *A. arguta* plants before flowering. It usually takes five to seven years for the *A. arguta* plant to get through its juvenile phase, and there is often higher survival rate for male seedlings than females^11^. Since the main purpose of kiwifruit breeding is to obtain female types with excellent fruit traits, the surplus of male individuals inevitably increases the costs associated with breeding, such as for land and management. The long juvenile phase and dioecy of *A. arguta* makes the crossbreeding of *A. arguta* plants time consuming and costly.

The adoption of molecular markers provides a more efficient method for traits identification, and it is commonly used in sex identification studies of many fruit plants. Genomics approaches are currently the most practical method for developing sex-linked molecular markers in plants and animals. In previous studies, the BSA (Bulked Segregant Analysis) method has been performed to develop two sex-linked SCAR markers in *A. chinensis*^6,12^, and in the development of sex-lined markers in other organisms such as *Pistacia vera* (pistachio)^13^, *Sargassum thunbergia* (a brown macroalgae)^14^, *Arapaima gigas* (an Amazonian freshwater fish)^15^, and *Phoenix dactylifera* (the date palm)^16^. In *Diospyros lotus* (diploid persimmon)^17–19^, whole-genome sequencing has been used to characterize the sex determination system and sex-identifying maker. Besides, SSR (simple sequence repeats) markers with high reliability have been developed in *Myrica rubra* (Chinese bayberry)^20^ and *A. chinensis*^7^. However, markers that provide high accuracy in *A. chinensis* are not universal in *A. arguta*^12,21^. Moreover, genotyping-by-sequencing (GBS) analysis was adopted to develop sex-linked molecular markers for *A. arguta* and *A. kolomikta* by a previous study, and the marker aC36306 has been used for sex-identifying of *A. arguta* seedlings^22^. However, the location of aC36306 in the genome is still uncertain to date, and these is no significant similarity of it found in the NCBI database.

In the present study, a whole-genome resequencing approach was adopted to develop molecular markers that could be applied to the early sex identification and help further research on the mechanism of sex determination of *A. arguta*. Additionally, the DNAs of *A. chinensis* genotypes was extracted to test the universality of the markers in interspecific populations. The two markers turned out to be highly reliable in sex identification of *A. arguta* plants and they were also of great value to cross-breeding programs of *A. arguta*.

## Materials and methods

### Plant material

For whole-genome resequencing, we randomly sampled 30 F1 individuals of known gender (15 females and 15 males) from the population of ‘HB’ (a cultivar of* A. arguta*, female) × ‘11–17’ (a line of *A. arguta*, male), grown at the Xinxiang Comprehensive Experimental Base of the Chinese Academy of Agricultural Sciences (CAAS). To test for the universality of the sex identification markers, 97 (52 females and 45 males) hybrid and 31 (18 males and 13 females) wild individuals were collected from the Zhengzhou Fruit Research Institute (ZFRI) of CAAS (Supplementary File 1). The wild individuals were introduced from public lands (i.e., no permission was required) in the Henan, Hubei and Guizhou provinces in China, and deposited at the ZFRI of CAAS. Additionally, 48 adult *A. chinensis* vines (16 males and 32 females) were used to verify the universality of the markers in *A. chinensis* Planch. Young leaves of the mature plants were harvested for genome DNA extraction.

### Illumina library construction and sequencing

The genomic DNA was extracted from young leaves using the Rapid Extraction Kit of the Beijing Labhelper Biotechnology Co. Ltd, with a slightly modified CTAB (cetyltrimethylammonium bromide) method, and purified using phenol/chloroform extraction. After quality testing, the genomic DNA from the same gender (15 males and 15 females) was used for the construction of two genome pools, and each pool represented an Illumina genomic library for whole-genome sequencing. The DNA was fragmented mechanically (ultrasonic method) and the fragments were purified for size selection. These selected fragments were enriched by PCR reaction to form a sequencing library. The enriched library was first subjected to library quality inspection and then the quality-qualified library was sequenced using Illumina HiSeq 2500 following the standard protocol provided by the manufacturer’s instructions.

### Sex-specific k-mer analysis

The clean reads obtained from the quality assessment of raw reads after filtering were used for subsequent bioinformatics analysis. To select sex-biased reads, the quality trimmed read files from both male and female pools were processed to identify gender-specific subsequences using Perl scripts. Then, a k-mer analysis of a high-quality sequence was performed to develop the candidate gender-specific reads for the assembly. We only selected 35 bp long k-mers starting with the “AG” dinucleotide from all reads to reduce the total number of k-mers and to optimize calculation speed and analysis time without affecting the gender identification. Besides, a set of subsequences that met a minimum total (male + female) count threshold of two and a maximum total count threshold of 200 for genomic k-mers were retained. The k-mer counts were then compared between male and female reads. Finally, pure male-specific k-mers (MSKs, with counts of zero in the female pool) were identified and used to extract the sex-biased reads from the original quality-trimmed reads sets based on the presence of at least one of the selected pure MSKs.

The reads obtained from the above-mentioned analysis were used as candidates for the male-specific double-end sequencing data and assembled using SOAPdenovo2^23^ with default parameters. Moreover, the original reads of both genetic pools were compared to the assembled sequence data obtained in the previous step by Bowtie^24^ (i.e., no mismatches were allowed). Based on the alignment analysis of the reads in the two pools, the assembly sequences were divided into two parts: the male-specific sequences and the unidentified sequences. The assembly sequences that had a sequencing depth only in the male pool were considered to be male-specific sequences. Those that did not meet this condition were collectively referred to as unidentified sequences. The sex-specific genes in the male-specific sequences were obtained using the online version of WebAUGUSTUS^25^.

### Screening and verification of sex-linked markers

The genomic DNA samples from 16 *A. arguta* vines (8 females and 8 males) were amplified by PCR with designed primers to obtained gender-specific candidate sequences. The PCR reaction consisted of 10 μL of 1 × Taq MasterMix (Dye), 400 nmol L^−1^ of the forward and reverse primers, and 20 ng of template DNA. The PCR conditions were as follows: pre-denaturation at 94 °C for 5 min; 94 °C for 30 s, 52–68 °C (52 °C, 53.1 °C, 55.2 °C, 58.2 °C, 61.9 °C, 64.9 °C, 67 °C, and 68 °C) 30 s renaturation, 72 °C for 40 s, over 35 cycles; and extension at 72 °C for 2 min. The amplified products were detected by 1% agarose gel electrophoresis. Finally, the accuracy of each pair of primers was verified in the rest of the *A. arguta* samples. The target fragments were extracted and purified using a TIANgel Midi Purification Kit (TIANGEN BIOTECH (Beijing, China) CO., LTD). The purified products were sequenced by Sanger.

## Results

### Identification of male-specific markers

**Figure 1:**
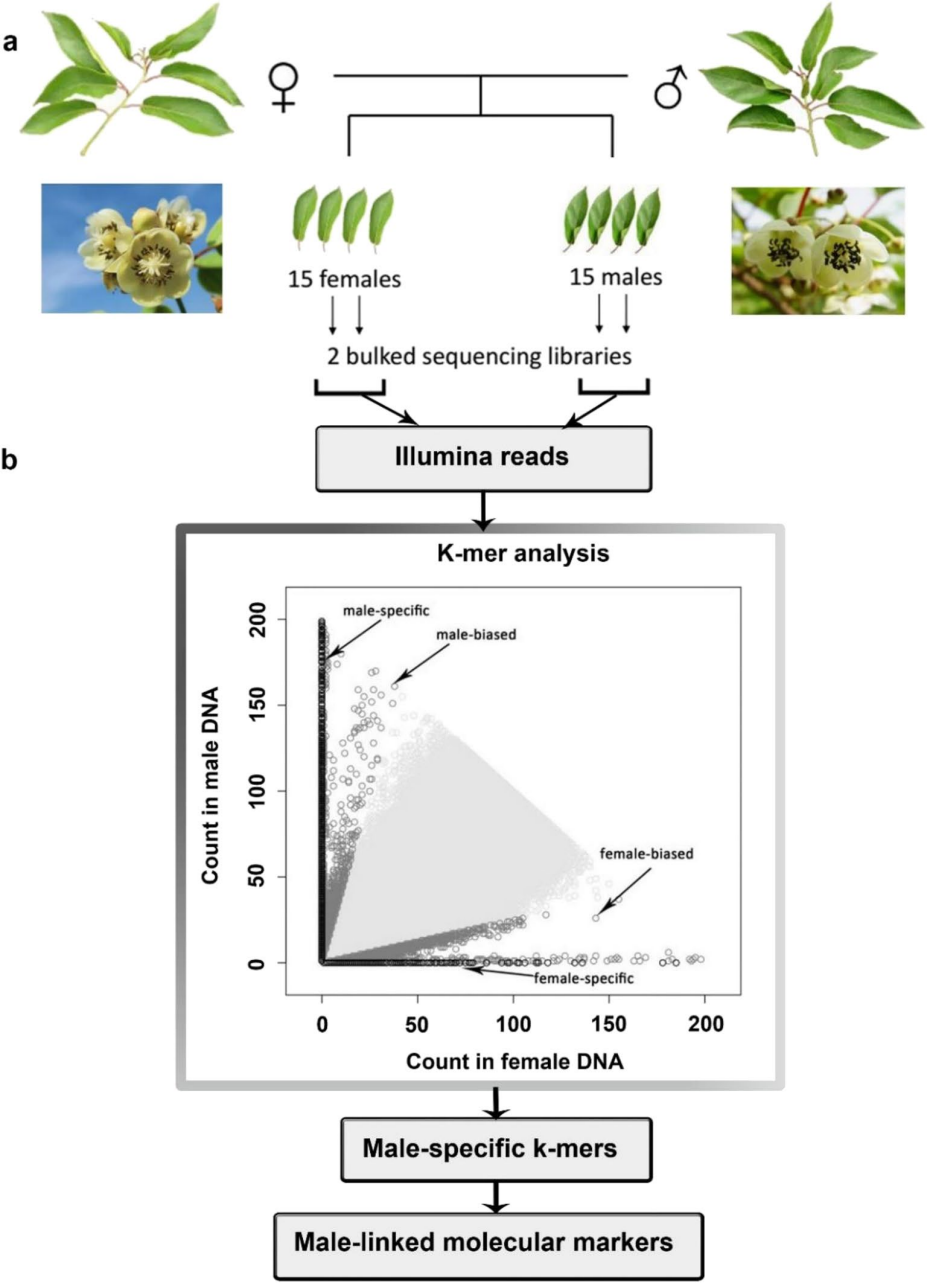
Identification of male-linked markers from genomic sequence reads. (**a**) The two genomic sequencing libraries were created from 15 females and 15 males from the F1 seedlings of ‘HB’ × ‘11–17’. The genome DNA was extracted from young leaves. (**b**) Reads from the two bulked pools were searched for the presence of gender-specific k-mers. The inset graph shows the distribution of gender-specific and gender-biased k-mers. The male-linked markers were identified from nine “male-specific” scaffolds obtained by the sex-specific k-mer analysis.

The two bulk segregant pools were constructed from 15 female and 15 male siblings of the F1 population (*A. arguta* cultivar ‘HB’, female × *A. arguta* male line ‘11–17’). The sequenced reads were obtained using Illumina HiSeq 2500 platform. We retrieved 30 Gb of clean data after filtering and screening. In total of 14,052,363 paired-end reads were extracted as candidate MSKs using Perl script. The De novo assembly of the genomic scaffolds was performed by the software SOAPdenove2 with all 150 bp paired-end (PE) reads including the MSKs (Fig. 1a). The candidate male-specific PE reads mentioned above were assembled into 5786 reads. The reads corresponding to the MSKs may have also been present in the female pool, meaning that both the male-specific reads and the non-sex-specific reads existed amongst the 5786 reads.

**Figure 2:**
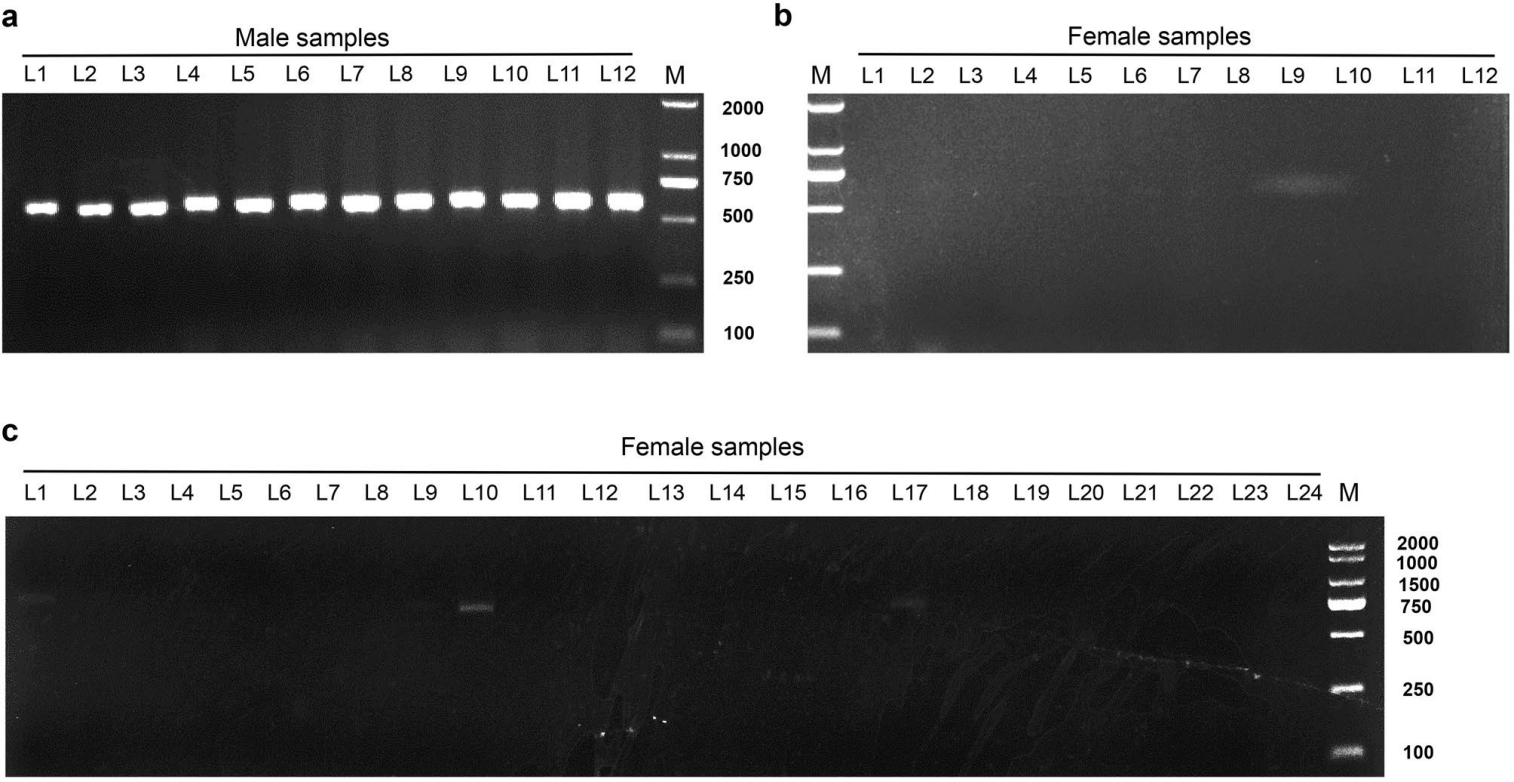
Amplification of 48 genomic DNAs from *A. arguta* by P51 at an annealing temperature of 60 °C. Amplification results of P51 in 12 male samples (**a**) and 12 female samples (**b**) from the population of ‘HB’ (a cultivar of *A. arguta*, female) × ‘11–17’ (a line of *A. arguta*, male). Amplification results of P51 in the other 24 female samples (**c**). Weak bands were detected in the female samples: L10 and L17 (**c**). The PCR amplification products were detected by 1% agarose gel electrophoresis. The size of the amplification product of P51 was 609 bp, as determined by Sanger sequencing (L = lane, different numbers mean different plant samples; M = 2000 bp DNA Marker).

**Figure 3:**
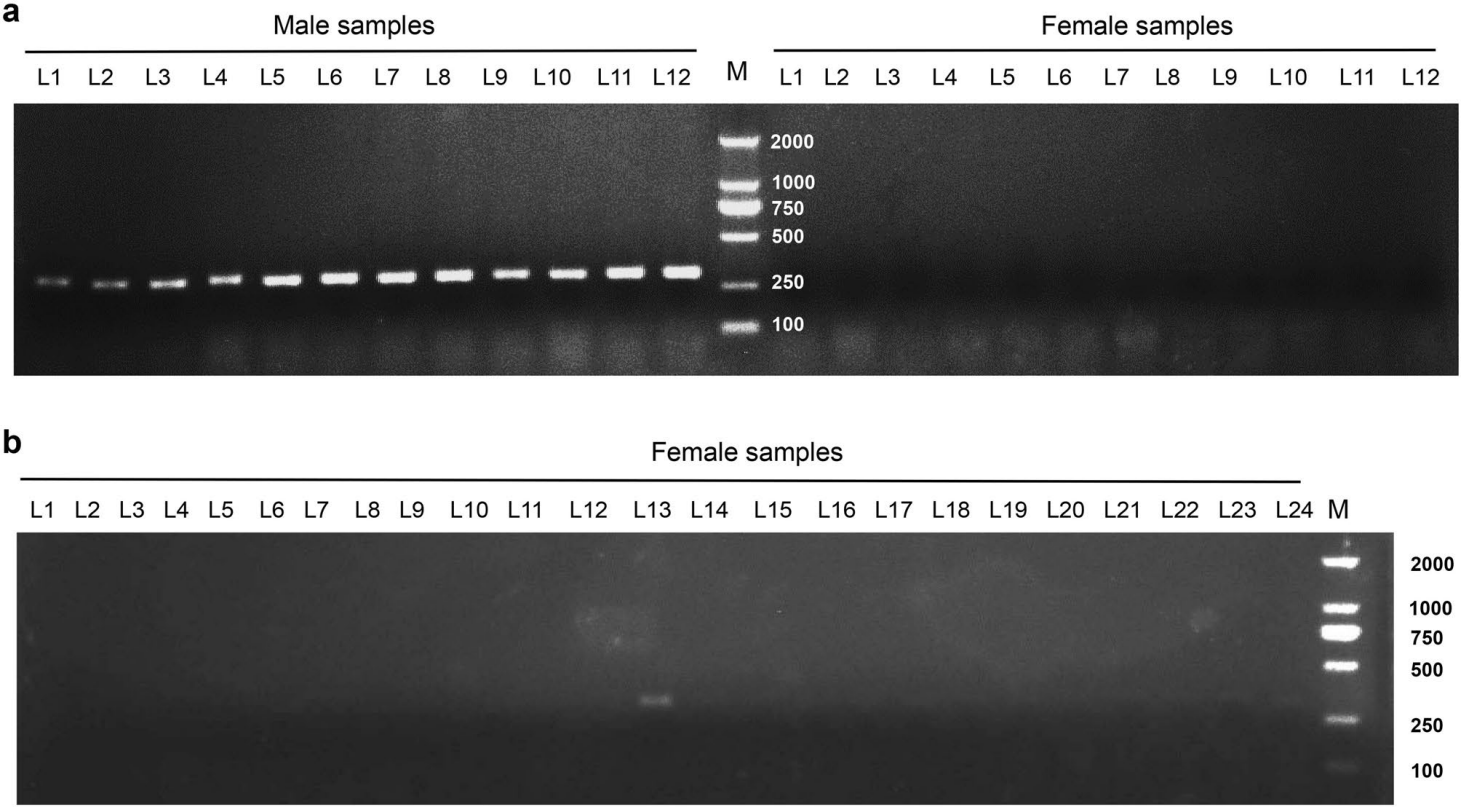
Amplification of 48 genomic DNAs from *A. arguta* by P11 at an annealing temperature of 65 °C. Amplification results of P11 in 12 male samples and 12 female samples (**a**) from the population of ‘HB’ (a cultivar of *A. arguta*, female) × ‘11–17’ (a line of *A. arguta*, male). Amplification results of P11 in the other 24 female samples (**b**). A weak band was detected in the female sample: L13 (**b**). The PCR amplification products were detected by 1% agarose gel electrophoresis. The size of the amplification product of P11 was 304 bp, as determined by Sanger sequencing (L = lane, different numbers mean different plant samples; M = 2000 bp DNA Marker).

The assembled k-mers were divided into two parts by the bowtie software: male-specific scaffolds and pendings. The male-specific scaffolds were defined by a complete lack of mapped reads from the female pool, and the other contigs were defined as “pending”. In this study, we identified 19 “male-specific” scaffolds. Additionally, we retained 19 other “male-specific” scaffolds from the pending scaffolds in the case when the coverage of the male pool was twice that of the female mixed pool (Fig. 1b). We identified nine sequences by analyzing 38 male-specific scaffolds, in WebAUGUSTUS, using default parameters settings (Supplemental Table S1). The primers were designed and verified using PCR for each of the 9 sequences. According to the results, only two pairs of primers, Primer 51 (P51) for sequence g5.t1 and Primer 11 (P11) for sequence g11.t2, proved to be sex-linked (Table 1 and Supplemental Table S1). These two pairs of primers only amplified a single band among males but not among females (Figs. 2 and 3).

**Table 1:** Overview of P51 and P11.

Primer name	Primer sequence (5′ → 3′)	Tm/℃	Band size	Marker type
P51	F: TCTTCCTCTTGGTGCCCG	60	609	Male
	R: TCAAAGAACCGCTAATCCCAT			Dominant
P11	F: TCTCGCCATCTTCAACCATTT	65	304	Male
	R: ATCTTGGCGTATCTCGGCTTT			Dominant

### Verification of the markers in *A. arguta* and *A. chinensis*

To verify the accuracy of the two markers in the *A. arguta* plants, 97 hybrids (52 females and 45 males) and 31 wild (13 females and 18 males) individuals, from the kiwifruit resource nursery of ZFRI at CAAS were sampled for PCR tests. According to the PCR results, both P51 and P11 could distinguish the gender of *A. arguta* vines efficaciously (Figs. 2 and 3). The P51 amplified a 609 bp fragment in male samples (Fig. 2a), but not in the females (Fig. 2b and c) at an annealing temperature of 60 °C. The accuracy rate was 96.88% for P51 in the 128 samples mentioned above (Table 2), whilst P11 produced a 304 bp fragment in male samples (Fig. 3a), but not in the females (Fig. 3a and b) at an annealing temperature of 65 °C, with an accuracy rate of 96.09% (Table 2). The two sequences L51 (the amplified products of Primer 51) and L11 (the amplified products of Primer 11) were sequenced using Sanger (Supplementary File 2).

**Table 2:** Accuracy rate of Primer 51 and Primer 11 in *Actinidia arguta*.

Sample	Resource	Primer 51		Primer 11	
		Presence	Absence	Presence	Absence
Female (52)	F1	2	50	2	50
Female (13)	Wild	1	12	2	11
Male (45)	F1	45	0	45	0
Male (18)	Wild	17	1	17	1
Accuracy rate		96.88%		96.09%	

F1 stands for the hybrid offsprings of ‘HB’ (a cultivar of *A. arguta*, female) × ‘11–17’ (a line of *A. arguta*, male); Wild means the samples were collected from wild places. The numbers after word ‘Female’ or ‘Male’ in the bracket represent sample numbers.

In addition, we tested the two sex-linked markers in 48 *A. chinensis* plants with known genders. The P51 marker did not indicate any sex-linked bands in *A. chinensis* samples. The results of P11 in *A. chinensis* showed a low accuracy rate: the specific bands could be amplified in all 16 male samples and some females. Therefore, these markers are not suitable for use in gender identification of *A. chinensis* plants.

## Discussion

In the present study, we used the de novo whole-genome sequencing technique to develop sex-identification markers in an *A. arguta* hybrid population of ‘HB’ and ‘11–17’. The P51 and P11 markers were proved to be sex-linked, and could potentially be used in the identification of *A. arguta* seedlings. In our preliminary tests, the products of both primers displayed bands of similar size in both the female and male samples, while there were usually two bands with almost the same sizes in males. Some previous studies described similar situations where bands of a similar sizes appeared in both males and females at lower annealing temperatures^12,26^. Nevertheless, gender-specific bands were only obtained in male samples at higher annealing temperatures^12^. Therefore, we set eight annealing temperature gradients from 52 to 68 °C for P11 and P51. At the 65 °C annealing temperature, the band for P11 appeared only in males. Likewise, for P51, we obtained the sex-specific band only in males at 60 °C annealing temperature.

According to the BLAST results of L51 and L11 in the GenBank (https://blast.ncbi.nlm.nih.gov/Blast), the two sequences were closely aligned with the Y male-specific region of *A. chinensis* genome and an *A. rufa* x *A. chinensis* Linkage map^9^. Additionally, L51 and L11 were similar to different parts of contig LC482709.1, which was located on the male-specific region of *A. chinensis* genome, and the sex determining gene *FrBy* was also located on contig LC482709.1 (Supplemental Fig. S1)^27,28^. Hence, we conjecture that these two markers are probably located in the Y-specific region of the sex chromosome. If this inference turns out to be correct, these markers may play a role in the localization of sex-determining loci. However, when BLAST was performed to identify the closest homolog of these two markers against the genome of *A. chinensis* (Hong Yang v2)^28^, the results showed that the target hits were on the chromosome 00, suggesting that the two alignments had not been scaffolded to any of the 29 chromosomes. Therefore, the exact positions of these two markers cannot be determined until a well-scaffolded genome of *A. arguta* is available.

After verification, the previously published sex-linked marker aC36306 for *A. arguta* can achieve 93.75% accuracy in 32 *A. arguta* plants (Supplementary file 6)^22^, and it has already been used in *A. arguta* seedlings. However, since the location of aC36306 in the genome is still uncertain to date, and these is no significant similarity of it found in the NCBI database, it is difficult to be applied to subsequent research on sex formation. Therefore, our work could be defined as an improvement to those results because of the diversity of the samples adopted in our study and the potential value for continuing research on the mechanism of sex determination.

In our present study, P51 and P11 were unable to accurately identify the gender of *A. chinensis* individuals. Similar situations also occurred in previous studies. The sex-linked molecular markers developed in *A. chinensis* could not identify the sex of *A. arguta*^21^. In *Diospyros*, sex-linked marker DlSx-AF4S developed in *Diospyros kaki* Thunb showed a high transferability in species related to *D. kaki* and *D. lotus*, but still failed to distinguish the gender of all the seedlings in other populations^19^. Additionally, in several other plants, such as the Chinese bayberry (*Myrica rubra*), papaya (*Carica papaya*), and pistachios (*Pistacia vera*)^20,29,30^, sex-linked markers have been known to show different accuracies for plants from the same genus but different species.

Hence, we speculate that the main reason for the poor universality of these markers is that the two species (*A. arguta* and *A. chinensis*) are not genetically close, even though they both belong to the genus *Actinidia*. Most subspecies of *A. chinensis* are diploid and tetraploid^31^, while subspecies of *A. arguta* are diploid, tetraploid, hexaploid, and octoploid^32^. The plant material from *A. chinensis* and *A. rufa* that was used to develop the three SSR markers was diploid^9^. Although, the sex chromosome of kiwifruit is reportedly chromosome 25, it has not evolved any obvious morphological feathers that differ from those of other euchromosomes^9,32^. It has also been reported that the sex-determining locus of kiwifruit is narrowed-down to a subtelomeric region of the sex chromosome^33^. The subtelomeric region of the chromosome is rich in satellites and lacks recombination, making it one of the most dynamic and rapidly evolving regions of the eukaryotic genomes^10^. Moreover, the developmental rule of kiwifruit during the gender differentiation process is very similar to that of asparagus, which is a representative plant of plants with sex chromosomes in the second stage of evolution^34–36^. Since the Y chromosome of kiwifruit is still in the early stage of evolution, and the genetic distance between *A. arguta* and *A. chinensis* is long, we speculate that the location and/or sequence of the sex-determination region of *A. arguta* may be different from that of *A. chinensis*. Either the loci of sex-determining region vary from species to species in *Actinidia* or the sequence is not conserved around the sex determination loci^37^.

Additionally, previous studies have shown that plant hormones, genetic factors, and epigenetic modifications may determine the sex of plants through mutual interactions, dynamic processes that are independent and synergistic, and diversification of the gender phenotypes^34^. Transcriptome research on *A. arguta* flowers from different genders revealed that the expression level of *PME* (pectin methylesterase gene) in the male flower was 72 folds higher than that in the female flower at the late stage of male-flower development^38^, but *PME* may not directly participate in the sex determination of dioecious plants^39–41^. In *A. Chinensis*, it was reported that two Y chromosome-encoded genes, *SyG1* and *FrBy*, located in the Y-specific region of chromosome 25, acted independently as factors controlling gender determination^27,34^. *SyG1* encodes a cytokinin response regulator, it is fully conserved in kiwifruit and can dominantly suppress the development of the carpel^34,42^. *SyGl* also affects the expression of genes involved in cytokinin metabolism and signaling, and the *SyGl* mutant can self-pollinate and produce offspring^43^. Furthermore, cytokinin has been previously proven that it has a strong induction effect on the formation of male gametophytes of *Blechnum spicant* (deer fern)^44^. On the other hand, on the basis of the evidence that the expression of *FrBy* in female kiwifruit resulted in hermaphrodite plants, the selective expression of *FrBy* in the early developing period of androecia has a high potential to regulate the degradation of tapetum via programmed cell death (PCD)^27,45^. It is believed that in the near future, the mechanism for gender differentiation of kiwifruit plants will be readily resolved.

## Conclusion

In this study, P51 and P11 distinguished the gender of *A. arguta* effectively but failed to identify the gender of *A. chinensis* samples, which indicates their universal application in *A. arguta* plants only. Consequently, these two markers are useful for sexual identification and thus, will accelerate the breeding process of *A. arguta*.

### Supplementary Information

Supplementary Figure S1.

Supplementary Tables.

Supplementary Information 3.

Supplementary Information 4.

Supplementary Information 5.

Supplementary Information 6.

Supplementary Information 7.

Supplementary Information 8.

## Data Availability

All sequence data generated in the context of this manuscript have been deposited in NCBI database (BioProject ID: PRJNA874682), and the details of the data are described in Supplementary file 4. URL: https://www.ncbi.nlm.nih.gov/bioproject/PRJNA874682/.
