# Supplementary material for: Development of sex-linked markers for gender identification of Actinidia arguta
Source: Sci Rep. 2023 Aug 7;13:12780. doi: 10.1038/s41598-023-39561-0 (PMC10406875; doi:10.1038/s41598-023-39561-0)
Supplement: Supplementary file 1 — Supplementary Figure S1. [file 41598_2023_39561_MOESM1_ESM.pdf]

Supplemental Figure

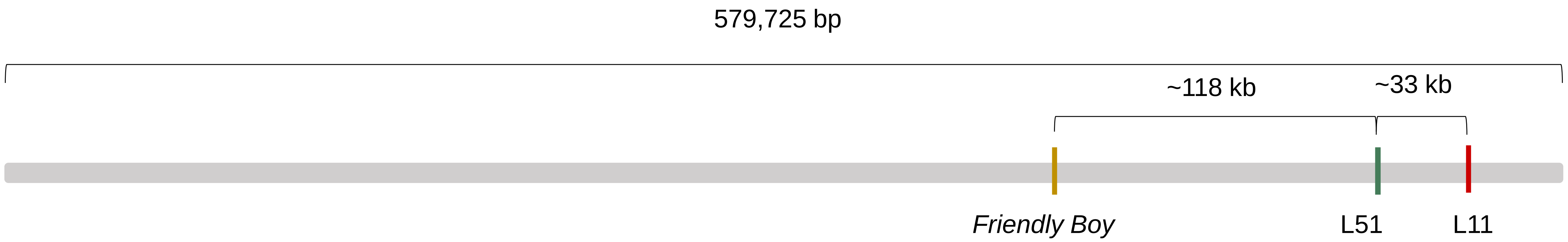

Figure S1. Putative physical loci of homology regions of L51 and L11 on male-specific region of *A. chinensis*

Note: L51 and L11 were aligned to different parts of LC482709.1, respectively. The male sex-determining gene *Friendly boy* of *A. chinensis* is also located on LC482709.1 (Akagi et al. 2019. Nature Plants.). The distance between L51 and L11 is ~33kb, and the distance between *Friendly boy* and L51 is ~118kb.
