# Supplementary material for: Development of sex-linked markers for gender identification of Actinidia arguta
Source: Sci Rep. 2023 Aug 7;13:12780. doi: 10.1038/s41598-023-39561-0 (PMC10406875; doi:10.1038/s41598-023-39561-0)
Supplement: Supplementary file 2 — Supplementary Tables. [file 41598_2023_39561_MOESM2_ESM.pdf]

Supplemental Table S1. Sex-different sequences and the primer sequences

Name	Primer name	Primer-F (5'→3')	Primer-R (5'→3')
**g11.t2**	**P11**	**TCTCGCCATCTTCAACCATTT**	**ATCTTGGCGTATCTCGGCTTT**
g1.t1	P1.1	CCTCTCCGAACTAAGGATTGC	CGCTACAAATCCCTCCGC
g11.t3	P11-3	CCTGAGGTGGGCATGTCGT	CCGTAGGCGAGGTGATGTTG
g2.t2	P2-2	GAGTATCTTGGTTCACTTCCCTT	AGTCCCTGTCCTCTTGAAGTTC
**g5.t1**	**P51**	**TCTTCCTCTTGGTGCCCG**	**TCAAAGAACCGCTAATCCCAT**
g3.t1	P3-1	GGGGCGTAGACATACTTGGAC	ATGTTGAGTCTTAGCTCGGCG
g6.t1	P6-1	AAGGAAGATGATTGGGAAGACC	TTCAGCCCTACTTTCATCATCC
g4.t1	P4-1	GGGGAGTTCAAGCACAAAGAGT	GAGGACCAAATGGAAAGAAGCAC
g11.t1	P11-1	CCTGAGGTGGGCATGTCGT	CGCTACAAATCCCTCCGC

Supplemental Table S2. hypothetical locations of L51 and L11 identified in the male specific contigs

gene id	gene name	Highest hit in the TAIR10 DB	
		gene ID	putative function
kiwi151209_m20_contig_9:g1.t1		AT2G26450.1	Plant invertase/pectin methylesterase
kiwi151209_m20_contig_25:g2.t1		AT5G24470.1	APRR5, PRR5 | pseudo-response regulator
kiwi151209_m20_contig_38:g3.t1		AT3G22980.1	Ribosomal protein S5/Elongation factor
kiwi151209_m20_contig_40:g4.t1		AT4G03100.1	Rho GTPase activating protein
kiwi151209_m20_contig_59:g5.t1		AT4G35785.5	RNA-binding (RRM/RBD/RNP motifs) family
kiwi151209_m20_contig_92:g6.t1		AT2G39480.1	PGP6 | P-glycoprotein 6
kiwi151209_m20_contig_98:g7.t1		AT1G64260.1	MuDR family transposase
kiwi151209_m20_contig_105:g8.t1		ATCG00490.1	RBCL | ribulose-bisphosphate carboxylase
kiwi151209_m20_contig_154:g9.t1		ATCG00480.1	ATPB, PB | ATP synthase subunit beta
kiwi151209_m20_contig_155:g10.t1		AT3G29010.1	Biotin/lipoate A/B protein ligase
kiwi151209_m20_contig_182:g11.t1		ATCG00480.1	ATPB, PB | ATP synthase subunit beta
kiwi151209_m20_contig_229:g12.t1		AT4G15720.1	Tetratricopeptide repeat (TPR)-like
kiwi151209_m20_contig_229:g13.t1		AT1G28327.1	unknown protein; Has 52
kiwi151209_m20_contig_248:g14.t1		AT1G68010.1	HPR, ATHPR1 | hydroxypyruvate reductase
kiwi151209_m20_contig_256:g15.t1		AT2G04940.1	scramblase-related
kiwi151209_m20_contig_263:g16.t1		ATMG00860.1	ORF158 | DNA/RNA polymerases superfamily
kiwi151209_m20_contig_267:g17.t1		AT4G29420.1	F-box/RNI-like superfamily protein
kiwi151209_m20_contig_322:g18.t1	L51	AT2G19650.1	Cysteine/Histidine-rich C1 domain
kiwi151209_m20_contig_338:g19.t1		AT1G42190.1	GAG/POL/ENV polyprotein
kiwi151209_m20_contig_408:g20.t1		AT4G04740.2	CPK23 | calcium-dependent protein kinase
kiwi151209_m20_contig_496:g21.t1		AT1G19835.2	Plant protein of unknown function
kiwi151209_m20_contig_524:g22.t1		AT2G15180.1	Zinc knuckle (CCHC-type) family
kiwi151209_m20_contig_545:g23.t1		AT5G51480.1	SKS2 | SKU5 similar 2
kiwi151209_m20_contig_545:g24.t1	*Shy Girl*	AT5G26594.1	ARR24, RR24 | response regulator 24
kiwi151209_m20_contig_578:g25.t1		AT3G57300.1	INO80, ATINO80 | INO80 ortholog
kiwi151209_m20_contig_621:g26.t1	*Friendly Boy*	AT1G30800.1	Fasciclin-like arabinogalactan family
kiwi151209_m20_contig_624:g27.t1		AT1G09640.2	Translation elongation factor EF1B
kiwi151209_m20_contig_679:g28.t1		AT2G46230.1	PIN domain-like family protein
kiwi151209_m20_contig_743:g29.t1		AT5G45130.1	ATRAB5A, ATRABF2A | Rab5-related gene
kiwi151209_m20_contig_930:g30.t1		AT5G53790.1	Protein of unknown function (DUF295)
kiwi151209_m20_contig_951:g31.t1		AT3G04240.1	SEC | Tetratricopeptide repeat (TPR)-like
kiwi151209_m20_contig_962:g32.t1		AT5G21326.1	Ca2+regulated serine-threonine protein
kiwi151209_m20_contig_1037:g33.t1		AT3G26730.1	RING/U-box superfamily protein

kiwi151209_m20_contig_1060:g34.t1		AT3G24255.1	RNA-directed DNA polymerase
kiwi151209_m20_contig_1070:g35.t1		AT1G58390.1	Disease resistance protein (CC-NBS-LRR)
kiwi151209_m20_contig_1092:g36.t1		AT2G31650.1	ATX1, SDG27 | homologue of trithorax
kiwi151209_m20_contig_1107:g37.t1		ATMG00860.1	ORF158 | DNA/RNA polymerases superfamily
kiwi151209_m20_contig_1148:g38.t1		AT5G15090.2	VDAC3 | voltage dependent anion channel
kiwi151209_m20_contig_1182:g39.t1		no hit	NA
kiwi151209_m20_contig_1217:g40.t1		AT3G20810.3	JMJD5 | 2-oxoglutarate (2OG) and Fe(II)-oxygenases
kiwi151209_m20_contig_1263:g41.t1		AT4G11730.1	Cation transporter/ E1-E2 ATPase family
kiwi151209_m20_contig_1434:g42.t1		no hit	NA
kiwi151209_m20_contig_1436:g43.t1		AT3G28790.1	Protein of unknown function (DUF1216)
kiwi151209_m20_contig_1494:g44.t1		AT5G61200.1	unknown protein
kiwi151209_m20_contig_1494:g45.t1		AT1G68990.1	MGP3 | male gametophyte defective 3
kiwi151209_m20_contig_1724:g46.t1	*YFT*	AT1G65480.1	FT | PEBP (phosphatidylethanolamine-binding)
kiwi151209_m20_contig_1854:g47.t1		AT3G13590.1	Cysteine/Histidine-rich C1 domain family
kiwi151209_m20_contig_1986:g48.t1		no hit	NA
kiwi151209_m20_contig_2004:g49.t1		AT3G12350.2	F-box family protein
kiwi151209_m20_contig_2011:g50.t1		AT3G63190.1	RRF, HFP108 | ribosome recycling factor
kiwi151209_m20_contig_2206:g51.t1		AT4G26900.1	AT-HF, HISN4 | HIS HF
kiwi151209_m20_contig_2243:g52.t1	L11	AT3G55950.1	CCR3, ATCRR3 | CRINKLY4 related 3
kiwi151209_m20_contig_2578:g53.t1		AT3G60980.1	Tetratricopeptide repeat (TPR)-like
kiwi151209_m20_contig_2616:g54.t1		AT5G51630.2	Disease resistance protein (TIR-NBS-LRR)
kiwi151209_m20_contig_2826:g55.t1		AT3G57280.1	Transmembrane proteins 14C
kiwi151209_m20_contig_2924:g56.t1		AT3G12250.4	TGA6, BZIP45 | TGACG motif-binding factor
kiwi151209_m20_contig_3379:g57.t1		AT4G19930.1	F-box and associated interaction domain
kiwi151209_m20_contig_3626:g58.t1		AT5G17520.1	RCP1, MEX1 | root cap 1 (RCP1)
kiwi151209_m20_contig_3804:g59.t1		AT1G70040.1	Protein of unknown function (DUF1163)
kiwi151209_m20_contig_3872:g60.t1		AT2G30500.2	Kinase interacting (KIP1-like) family
kiwi151209_m20_contig_3940:g61.t1		AT4G03020.2	transducin family protein / WD-40 repeat

Note: the dataset is from Akagi, T. et al. (2019). Nature Plants https://doi.org/10.1038/s41477-019-0489-6
