# Supplementary material for: Development of sex-linked markers for gender identification of Actinidia arguta
Source: Sci Rep. 2023 Aug 7;13:12780. doi: 10.1038/s41598-023-39561-0 (PMC10406875; doi:10.1038/s41598-023-39561-0)
Supplement: Supplementary file 3 — Supplementary Information 3. [file 41598_2023_39561_MOESM3_ESM.pdf]

Development of Sex-linked Markers for Gender Identification of Actinidia arguta

Yunpeng Zhong†, Dandan Guo†, Ran Wang, Xiujuan Qi* & Jinbao Fang*

Key Laboratory for Fruit Tree Growth, Development and Quality Control, Zhengzhou Fruit Research Institute, Chinese Academy of Agricultural Sciences, Zhengzhou 450009, China.

† These two authors contributed equally: Yun-peng Zhong, Dan-dan Guo.

Correspondences: Xiujuan Qi, E-mail: qixiujuan@caas.cn; Jinbao Fang, E-mail: fangjinbao@caas.cn

Supplemental information

Statements of plant materials

In this study, we used four types of plant materials:

(1) 30 F1 individuals with known gender, 15 females and 15 males, from the population of 'HB' (a cultivar of *Actindia arguta*, female) × '11-17' (a line of *A. arguta*, male). 'HB' and '11-17' were collected in Luoyang city of Henan province in China, and planted in ZFRI CAAS. When we got the hybrid population, the population were planted in Xinxiang Comprehensive Experimental Base of CAAS (Chinese Academy of Agricultural Sciences);

(2) 97 individuals, 52 females and 45 males, from the same hybrid population of 'HB'×'11-17';

(3) 31 wild individuals, 18 males and 13 females, were collected from from Henan, Hubei and Guizhou provinces in China, and were kept in ZFRI CAAS (Zhengzhou Fruit Research Institute, Chinese Academy of Agricultural Sciences); (4) 48 adult *A. chinensis*,16 males and 32 females, were kept in ZFRI CAAS.

These materials are self owned and have no disputes with other units or individuals.

Statements of ‘HB’

‘HB’ is one cultivated variety from *A. arguta*, named as ‘Zhongxiahong’, abbreviated as ‘HB’ because of the red (in chinese means **Hong**) flesh, and *A. arguta* is also called kiwi**b**erry. The application for new plant variety right of ‘HB’ is in progress. In China, it takes at least three years to get the certificate. The following is the application materials of ‘HB’ from Ministry of Agriculture and Rural Areas of China, and the application No. is 20191005940.

品种权申请请求书英文信息表

1 品种暂定名称(中英文)<br>仲夏红 Zhong Xia Hong		
2 培育人<br>Fang Jinbao, Qi Xiujuan, Chen Jinyong, Lin Miaomiao, Gu Hong, Sun Leiming, Zhong Yunpeng, Wang Ran		
3 申请人	① 代表	名称或姓名：Zhengzhou Fruit Research Institute, Chinese Academy of Agricultural Sciences<br>地址：The South End of Weilai Road, Zhengzhou, Henan, PR. China,
	②	名称或姓名：<br>地址：
	③	名称或姓名：<br>地址：

MARA.CNPVP

20191005940

Statements of wild germplasm resources

In this study, 31 wild individuals of *Actinidia arguta*, 18 males and 13 females, were used to check the accuracy of two primers, and collected from Henan, Hubei and Guizhou provinces in China. After collecting, these wild resources were top-grafted and kept in ZFRI CAAS (Zhengzhou Fruit Research Institute, Chinese Academy of Agricultural Sciences).

The detailed informations of wild germplasm resources used in this article were as follows:

Number	Gender	Collection site, City/Province	Geographical coordinates	Altitude/m	Collector	Collection time
01	Female	Jiangkou County, Tongren City, Guizhou Province	E 108°79′04″, N 27°72′84″	492	Xiujuan Qi, Yunpeng Zhong	2017.9
02	Female	Jiangkou County, Tongren City, Guizhou Province	E 108°78′03″, N 27°73′14″	569	Xiujuan Qi, Yunpeng Zhong	2017.9
03	Female	Jiangkou County, Tongren City, Guizhou Province	E 108°77′47″, N 27°73′07″	613	Xiujuan Qi, Yunpeng Zhong	2017.9
04	Female	Jiangkou County, Tongren City, Guizhou Province	E 108°78′29″, N 27°74′45″	582	Xiujuan Qi, Yunpeng Zhong	2017.9
05	Male	Jiangkou County, Tongren City, Guizhou Province	E 108°78′54″, N 27°75′26″	526	Xiujuan Qi, Yunpeng Zhong	2017.9
06	Male	Jiangkou County, Tongren City, Guizhou Province	E 108°77′54″, N 27°76′26″	626	Xiujuan Qi, Yunpeng Zhong	2017.9
07	Male	Jiangkou County, Tongren City, Guizhou Province	E 108°78′54″, N 27°77′26″	628	Xiujuan Qi, Yunpeng Zhong	2017.9
08	Male	Jiangkou County, Tongren City, Guizhou Province	E 108°79′53″, N 27°75′22″	685	Xiujuan Qi, Yunpeng Zhong	2017.9

09	Male	Jiangkou County, Tongren City, Guizhou Province	E 108°79′51″, N 27°76′27″	677	Xiujuan Qi, Yunpeng Zhong	2017.9
10	Female	Tujia Autonomous County, Yichang City, Hubei Province	E 111°06′08″, N 30°16′34″	623	Xiujuan Qi, Yunpeng Zhong	2016.9
11	Female	Tujia Autonomous County, Yichang City, Hubei Province	E 111°08′35″, N 30°16′97″	705	Xiujuan Qi, Yunpeng Zhong	2016.9
12	Female	Tujia Autonomous County, Yichang City, Hubei Province	E 111°06′15″, N 30°15′27″	453	Xiujuan Qi, Yunpeng Zhong	2016.9
13	Male	Tujia Autonomous County, Yichang City, Hubei Province	E 111°07′17″, N 30°15′87″	558	Xiujuan Qi, Yunpeng Zhong	2016.9
14	Male	Tujia Autonomous County, Yichang City, Hubei Province	E 111°06′27″, N 30°14′63″	687	Xiujuan Qi, Yunpeng Zhong	2016.9
15	Male	Tujia Autonomous County, Yichang City, Hubei Province	E 111°05′18″, N 30°15′64″	762	Xiujuan Qi, Yunpeng Zhong	2016.9
16	Male	Tujia Autonomous County, Yichang City, Hubei Province	E 111°06′28″, N 30°16′54″	805	Xiujuan Qi, Yunpeng Zhong	2016.9
17	Male	Tujia Autonomous County, Yichang City, Hubei Province	E 111°06′48″, N 30°15′44″	658	Xiujuan Qi, Yunpeng Zhong	2016.9
18	Male	Tujia Autonomous County, Yichang City, Hubei Province	E 111°05′48″, N 30°19′34″	798	Xiujuan Qi, Yunpeng Zhong	2016.9
19	Male	Tujia Autonomous County, Yichang City, Hubei Province	E 111°06′58″, N 30°19′64″	835	Xiujuan Qi, Yunpeng Zhong	2016.9
20	Male	Tujia Autonomous County, Yichang City, Hubei Province	E 111°06′58″, N 30°18′47″	817	Xiujuan Qi, Yunpeng Zhong	2016.9

21	Female	Luanchuan County, Luoyang City, Henan Province	E 111°57′63″, N 33°95′18	1028	Xiujuan Qi	2014.8
22	Female	Luanchuan County, Luoyang City, Henan Province	E 111°57′63″, N 33°95′18	1027	Xiujuan Qi	2014.8
23	Female	Luanchuan County, Luoyang City, Henan Province	E 111°57′64″, N 33°95′19	824	Xiujuan Qi	2014.8
24	Female	Luanchuan County, Luoyang City, Henan Province	E 111°57′62″, N 33°95′28	928	Xiujuan Qi	2014.8
25	Female	Luanchuan County, Luoyang City, Henan Province	E 111°58′12″, N 33°95′16	768	Xiujuan Qi	2014.8
26	Female	Luanchuan County, Luoyang City, Henan Province	E 111°58′18″, N 33°95′37	1098	Xiujuan Qi	2014.8
27	Male	Luanchuan County, Luoyang City, Henan Province	E 111°56′07″, N 33°95′18	908	Xiujuan Qi	2014.8
28	Male	Luanchuan County, Luoyang City, Henan Province	E 111°57′83″, N 33°95′71	858	Xiujuan Qi	2014.8
29	Male	Luanchuan County, Luoyang City, Henan Province	E 111°59′92″, N 33°95′53	921	Xiujuan Qi	2014.8
30	Male	Luanchuan County, Luoyang City, Henan Province	E 111°61′76″, N 33°95′21	802	Xiujuan Qi	2014.8
31	Male	Luanchuan County, Luoyang City, Henan Province	E 111°60′26″, N 33°95′52	964	Xiujuan Qi	2014.8
