# Supplementary material for: Development of sex-linked markers for gender identification of Actinidia arguta
Source: Sci Rep. 2023 Aug 7;13:12780. doi: 10.1038/s41598-023-39561-0 (PMC10406875; doi:10.1038/s41598-023-39561-0)
Supplement: Supplementary file 4 — Supplementary Information 4. [file 41598_2023_39561_MOESM4_ESM.pdf]

Development of Sex-linked Markers for Gender Identification of Actinidia arguta

Yunpeng Zhong†, Dandan Guo†, Ran Wang, Xiujuan Qi* & Jinbao Fang*

Key Laboratory for Fruit Tree Growth, Development and Quality Control, Zhengzhou Fruit Research Institute, Chinese Academy of Agricultural Sciences, Zhengzhou 450009, China.

† These two authors contributed equally: Yun-peng Zhong, Dan-dan Guo.

Correspondences: Xiujuan Qi, E-mail: qixiujuan@caas.cn; Jinbao Fang, E-mail: fangjinbao@caas.cn

Supplemental information

L51-609bp: PCR amplified sequence of primer P51

TCTTCCTCTTGGTGCCCGGTGTTTCCCCATGGGGTATATGAATTTCCACTTGAA
GATCCGGAACTCTGACGCTTTGAATTTTGTCCTCTACTTGGACTCCCTCCAGAT
CGCTTCTTATTGCTCTCCCGATATTGGGACATCTCATCTAATGCTCTCTCAAAA
ATTATTGCTCATTGAAGGACCTCTTGATGGTTTGGCAATCGTAATATGTCTACT
TTGTTGTGGATGTTTCACTACAACCCTACTTCAAACTTTCTCACCTTGCGAGAT
TTTGTCAAAATAATATGCGGGGCATAACAAGATAATTCTATGAACTTGGCATT
GTACTCTACTACAGTCGTATTTCCTTGCACCAAATTAATAAATTCTACAATTTA
CTGATCCCTGACAGTTTCGAGAAAATACTCTTTGTTGAACACTTCTAAAACCCT
AGGCCACAACCATAACGGCTCCAGTAGCTTTTTCAACTACCACCAAACTAGTG
CGGCTTCCTCAAAAGTAAAGGTGGCAAGCGTCCCTTTCTGGTCATCAGTGCAA
GGTAGGACTTCGAACACTCTCTCAAATCCCAAGAGCCAAGATTCCGCGGTCAT
GGGATTAGCGGTTCTTTGA

L11-304bp: PCR amplified sequence of primer P11

TCTCGCCATCTTCAACCATTTCTCCTCGCCTGGTTATCCTCACGCAAATGGTCA
AGTAGACGTCACCGATAGAACTATCCTAAGAACTCTAAAGGTGAGGTTAGAAA
AGTCTAAGGGTGAATGGGCATAAGACCTTCAGATCATGCTATGGGATTACCAC
ACGACAAGCAGAATTCCTACAGGTGAGATGCCGTATTCTATGGTATATAGGAC
AGAATCAATCATACTTGTGGAGATAGGAATGCCGAGCTTCAAGACCTCAAATT
TTGACAAGGAGAACAACAAAGCCGAGATACGCCAAGAT
