# Supplementary material for: Development of sex-linked markers for gender identification of Actinidia arguta
Source: Sci Rep. 2023 Aug 7;13:12780. doi: 10.1038/s41598-023-39561-0 (PMC10406875; doi:10.1038/s41598-023-39561-0)

The original image of Fig 2

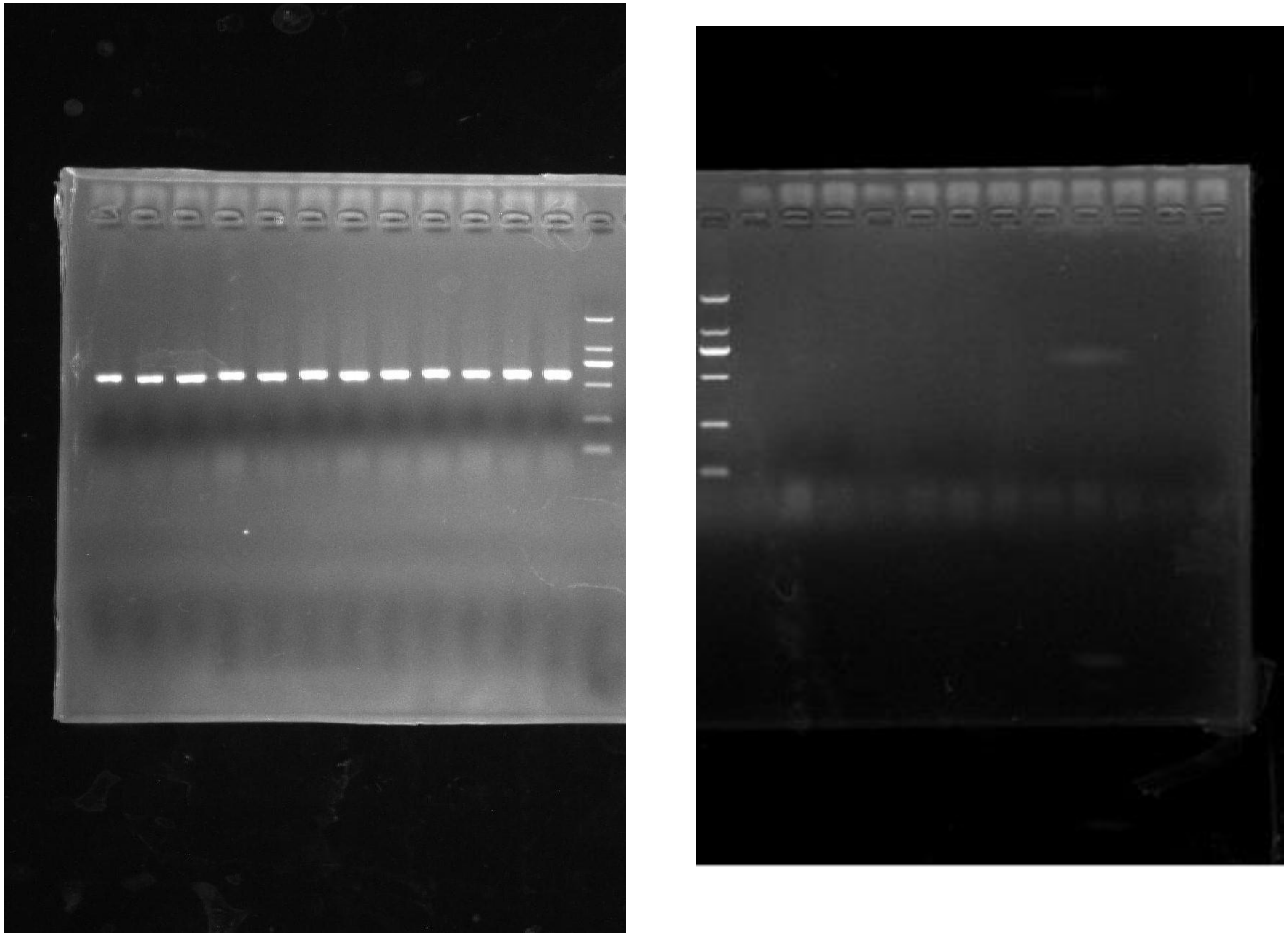

The original image of Fig 2

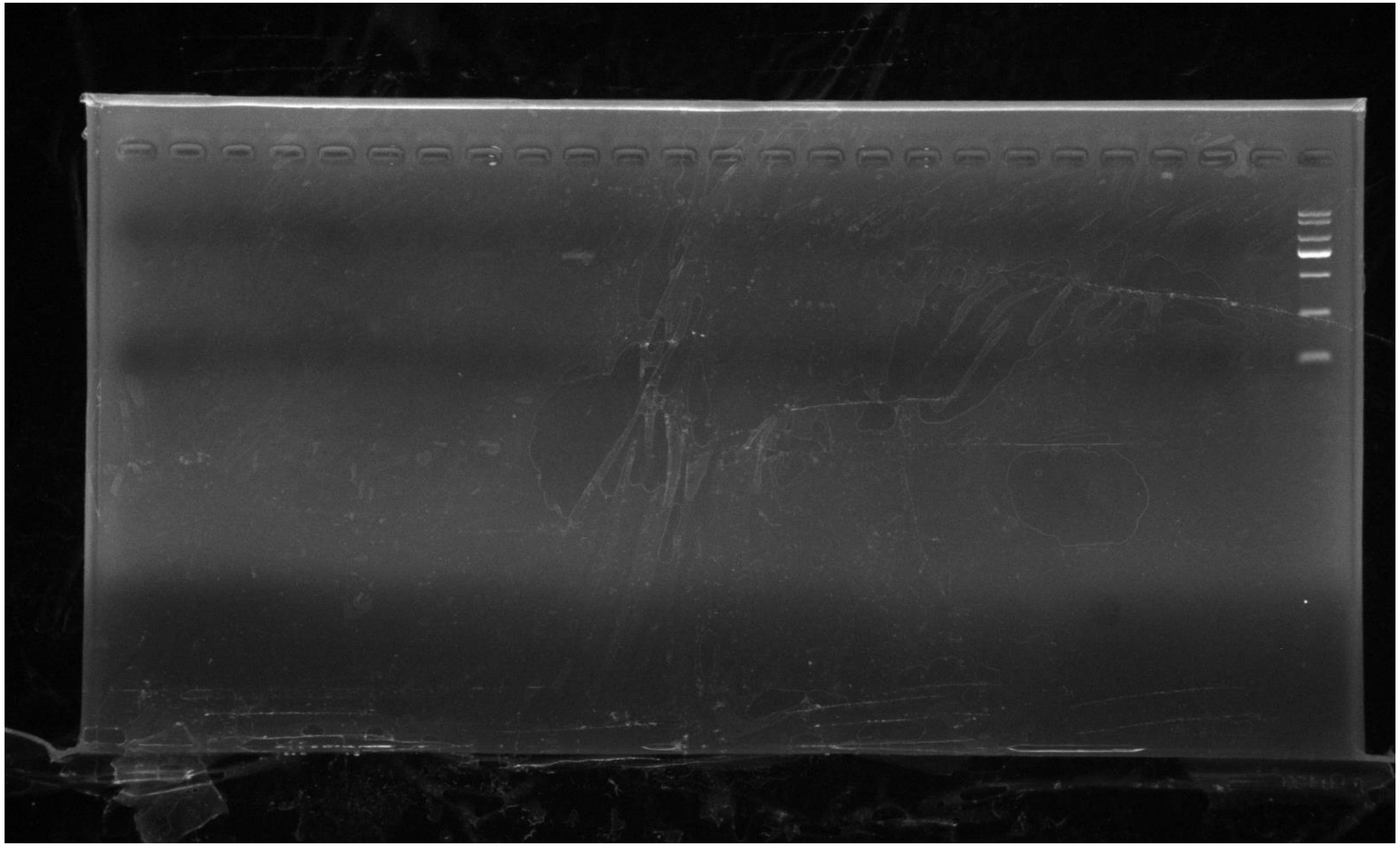

The original image of Fig 3

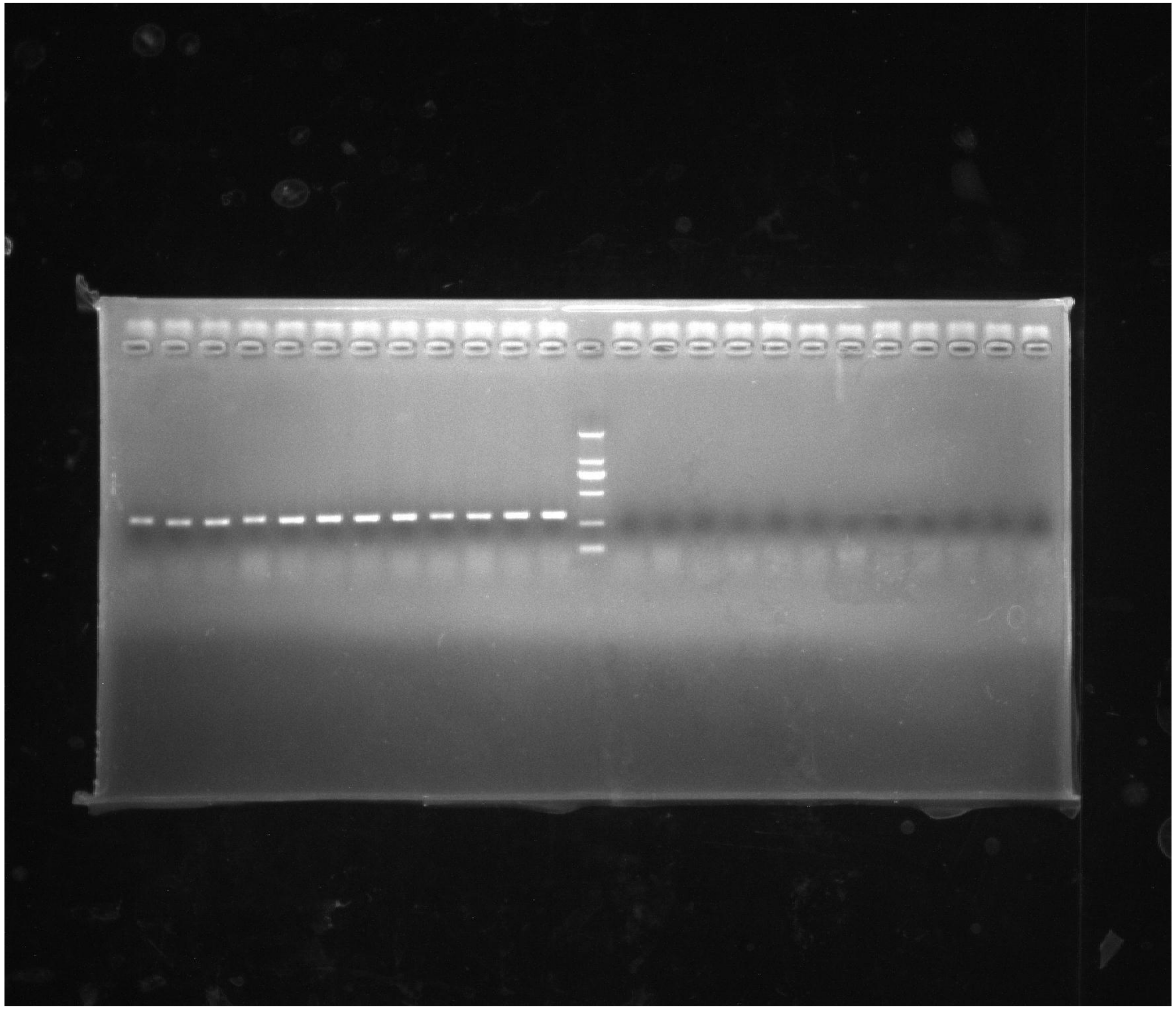

The original image of Fig 3

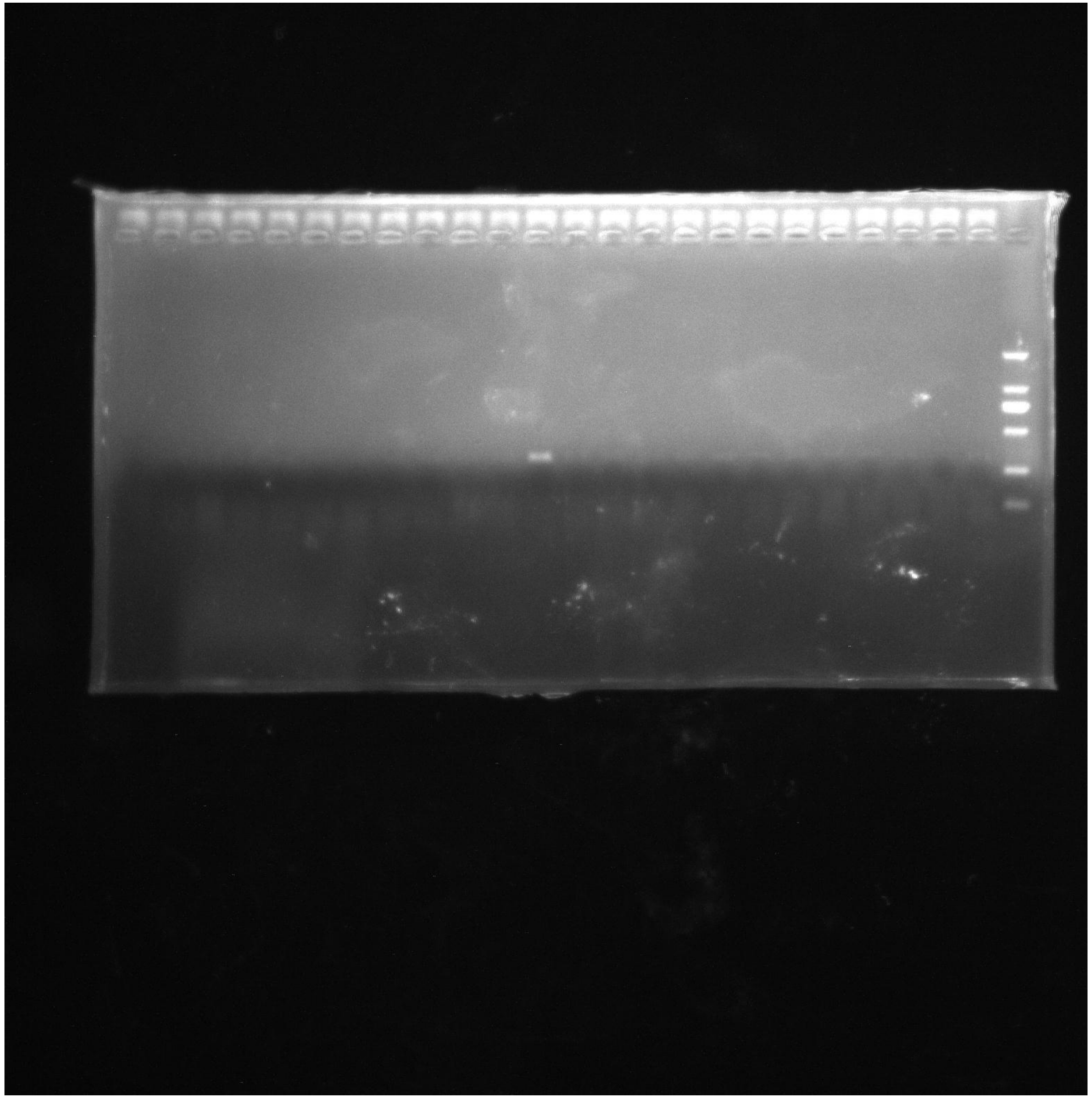

Supplement: Supplementary file 5 — Supplementary Information 5. [file 41598_2023_39561_MOESM5_ESM.pdf]
