# Supplementary material for: Development of sex-linked markers for gender identification of Actinidia arguta
Source: Sci Rep. 2023 Aug 7;13:12780. doi: 10.1038/s41598-023-39561-0 (PMC10406875; doi:10.1038/s41598-023-39561-0)
Supplement: Supplementary file 7 — Supplementary Information 7. [file 41598_2023_39561_MOESM7_ESM.docx]

#!/usr/bin/perl -w

use strict;

use warnings;

use Getopt::Long;

use Data::Dumper;

use Statistics::Distributions;

use FindBin qw($Bin $Script);

use File::Basename qw(basename dirname);

my $BEGIN_TIME=time();

my $version="1.0.0";

#######################################################################################

# ------------------------------------------------------------------

# GetOptions

# ------------------------------------------------------------------

my ($fIn,$t1,$t2,$sample,$fOut,$sw,$key);

GetOptions(

"help|?" =>\&USAGE,

"od:s"=>\$fOut,

"i:s"=>\$fIn,

"t1:s"=>\$t1,

"t2:s"=>\$t2,

"sample:s"=>\$sample,

"sw:s"=>\$sw,

"k:s"=>\$key,

) or &USAGE;

&USAGE unless ($fIn and $fOut);

$sw||=3;

mkdir $fOut if (!-d $fOut);

$t1||="0.66:0.33";

$t2||="0.01:0.99";

$sample||="P,aa,M,ab";

my @sample=split(/\,/,$sample);

my ($s1,$s2,$s3,$s4);

if (scalar @sample != 4) {

$sw=1;

}

if ($sw==3 || $sw==4) {

$s1=join("_",$sample[0],$sample[1]);

$s2=join("_",$sample[2],$sample[1]);

$s3=join("_",$sample[0],$sample[3]);

$s4=join("_",$sample[2],$sample[3]);

}

if ($sw==2) {

$s1="";

$s2="";

$s3=join("_",$sample[0],$sample[3]);

$s4=join("_",$sample[2],$sample[3]);

}

if ($sw==1) {

$s1=join("_",$sample[0],$sample[1]);

$s2=join("_",$sample[2],$sample[1]);

$s3="";

$s4="";

}

my $segregation;

my $theoretical_segregation;

open IN,$fIn or die $!;

my $title=<IN>;

$title=~s/\n//;

my @Indi;

my (@info,@p_value);

{

my ($MarkerID,$chr,$start,$end,$orientation,$parent);

($MarkerID,$chr,$start,$end,$orientation,$parent,@Indi)=split /\t/,$title;

if ($sw==4 ) {

push @info,"$MarkerID\t$chr\t$start\t$end\t$orientation\t$parent\t$s1\t$s2\t$s3\t$s4\tX2_"."$sample[1]\tp-value_"."$sample[1]\tX2_"."$sample[3]\tp_value_"."$sample[3]";

}

elsif ($sw==3){

push @info,"$MarkerID\t$chr\t$start\t$end\t$orientation\t$parent\t$s1\t$s2\t$s3\t$s4\tX2\tp-value";

}

elsif ($sw==2) {

push @info,"$MarkerID\t$chr\t$start\t$end\t$orientation\t$parent\t$s3\t$s4\tX2\tp-value";

}

elsif ($sw==1) {

push @info,"$MarkerID\t$chr\t$start\t$end\t$orientation\t$parent\t$s1\t$s2\tX2\tp-value";

}

}

while (<IN>) {

chomp;

next if (/^$/);

my ($MarkerID,$chr,$start,$end,$orientation,$parent,@indi)=split /\t/,$_;

if ($sw==4) {#sw=4

my %stat;

for (my $i=0;$i<@indi;$i++) {

$stat{$Indi[$i]}=$indi[$i];

}

my $seg1="$stat{$s1}:$stat{$s2}";

my ($d1,$d2)=split(/\:/,$t1);

if ($stat{$s1}+$stat{$s2}==0 || $stat{$s3}+$stat{$s4}==0) {

next;

}

my $thr1=join(":",$d1*($stat{$s1}+$stat{$s2}),$d2*($stat{$s1}+$stat{$s2}));

my $seg2="$stat{$s3}:$stat{$s4}";

my ($d3,$d4)=split(/\:/,$t2);

my $thr2=join(":",$d3*($stat{$s3}+$stat{$s4}),$d4*($stat{$s3}+$stat{$s4}));

my $X1=Segregation($thr1,$seg1);

my $X2=Segregation($thr2,$seg2);

my $info="$MarkerID\t$chr\t$start\t$end\t$orientation\t$parent\t$stat{$s1}\t$stat{$s2}\t$stat{$s3}\t$stat{$s4}\t"."$X1\t$X2";

push @info,$info;

push @{$p_value[1]},(split(/\s+/,$X1))[-1];

push @{$p_value[2]},(split(/\s+/,$X2))[-1];

}

elsif ($sw==3) {#sw=3

my %stat;

for (my $i=0;$i<@indi;$i++) {

$stat{$Indi[$i]}=$indi[$i];

}

my $seg1="$stat{$s1}:$stat{$s2}:$stat{$s3}:$stat{$s4}";

my ($d1,$d2,$d3,$d4)=split(/\:/,$t1.":".$t2);

my $thr1=join(":",$d1*($stat{$s1}+$stat{$s2}),$d2*($stat{$s1}+$stat{$s2}),$d3*($stat{$s3}+$stat{$s4}),$d4*($stat{$s3}+$stat{$s4}));

if ($stat{$s1}+$stat{$s2}==0 || $stat{$s3}+$stat{$s4}==0) {

next;

}

my $X1=Segregation($thr1,$seg1);

my $info="$MarkerID\t$chr\t$start\t$end\t$orientation\t$parent\t$stat{$s1}\t$stat{$s2}\t$stat{$s3}\t$stat{$s4}\t"."$X1";

push @p_value,(split(/\s+/,$X1))[-1];

push @info,$info;

}

elsif ($sw==2){#sw=2

my %stat;

for (my $i=0;$i<@indi;$i++) {

$stat{$Indi[$i]}=$indi[$i];

}

my $seg1="$stat{$s3}:$stat{$s4}";

my ($d3,$d4)=split(/\:/,$t2);

if ($stat{$s3}+$stat{$s4}==0) {

next;

}

my $thr1=join(":",$d3*($stat{$s3}+$stat{$s4}),$d4*($stat{$s3}+$stat{$s4}));

my $X1=Segregation($thr1,$seg1);

my $info="$MarkerID\t$chr\t$start\t$end\t$orientation\t$parent\t$stat{$s3}\t$stat{$s4}\t"."$X1";

push @p_value,(split(/\s+/,$X1))[-1];

push @info,$info;

}

elsif ($sw==1){#sw=1

my %stat;

for (my $i=0;$i<@indi;$i++) {

$stat{$Indi[$i]}=$indi[$i];

}

my $seg1="$stat{$s1}:$stat{$s2}";

my ($d1,$d2)=split(/\:/,$t1);

if ($stat{$s1}+$stat{$s2}==0 ) {

next;

}

my $thr1=join(":",$d1*($stat{$s1}+$stat{$s2}),$d2*($stat{$s1}+$stat{$s2}));

my $X1=Segregation($thr1,$seg1);

my $info="$MarkerID\t$chr\t$start\t$end\t$orientation\t$parent\t$stat{$s1}\t$stat{$s2}\t"."$X1";

push @info,$info;

push @p_value,(split(/\s+/,$X1))[-1];

}

}

close IN;

open RS,">$fOut/R.sh";

my $n1;

if ($sw==4) {

$n1=scalar @{$p_value[1]};

print RS "p_value1<-array(1:$n1)\n";

print RS "p_value2<-array(1:$n1)\n";

print RS "FDR1_out<-array(1:$n1)\n";

print RS "FDR2_out<-array(1:$n1)\n";

}

else {

$n1=scalar @p_value;

print RS "p_value<-array(1:$n1)\n";

print RS "FDR_out<-array(1:$n1)\n";

}

my $n=scalar @info;

print RS "info<-array(1:$n)\n";

for (my $i=0;$i<@info ;$i++) {

print RS "info[",$i+1,"]<- \"$info[$i]\"\n";

}

if ($sw==4) {

for (my $i=0;$i<@{$p_value[1]} ;$i++) {

print RS "p_value1[",$i+1,"]<-".$p_value[1][$i]."\n";

print RS "p_value2[",$i+1,"]<-".$p_value[2][$i]."\n";

}

print RS "FDR1<-p.adjust(p_value1,method=\"fdr\",n=$n1)\n";

print RS "FDR2<-p.adjust(p_value2,method=\"fdr\",n=$n1)\n";

for (my $i=0;$i<@{$p_value[1]} ;$i++) {

print RS "FDR1_out[",$i+2,"]<-FDR1[",$i+1,"]","\n";

print RS "FDR2_out[",$i+2,"]<-FDR2[",$i+1,"]","\n";

}

print RS "FDR1_out[",1,"]<-\"FDR_"."$sample[1]\"\n";

print RS "FDR2_out[",1,"]<-\"FDR_"."$sample[3]\"\n";

print RS "df<-data.frame(info,FDR1_out,FDR2_out)\n";

}

else {

for (my $i=0;$i<@p_value ;$i++) {

print RS "p_value[",$i+1,"]<-$p_value[$i]\n";

}

print RS "FDR<-p.adjust(p_value,method=\"fdr\",n=$n1)\n";

for (my $i=0;$i<@p_value ;$i++) {

print RS "FDR_out[",$i+2,"]<-FDR[",$i+1,"]","\n";

}

print RS "FDR_out[",1,"]<-\"FDR\"\n";

print RS "df<-data.frame(info,FDR_out)\n";

}

print RS "write.table(df,\"$fOut/$key.chi_test.txt\",row.names=FALSE,quote = FALSE,col.names=FALSE,sep = \"\\t\")","\n";

close RS;

`Rscript $fOut/R.sh`;

##################################################################################

sub Segregation {#

my ($theoretical_segregation,$segregation)=@_;

my @a=split ":",$theoretical_segregation;

my @b=split ":",$segregation;

return "-" if (scalar @a != scalar @b ) ;

my $df=scalar @a -1;

my $X2=0;

if ($df == 1) {

for (my $i=0;$i<@a ;$i++) {

$X2+=X2df2($b[$i],$a[$i]);

}

}else{

for (my $i=0;$i<@a ;$i++) {

$X2+=X2df1($b[$i],$a[$i]);

}

}

my $p_value=Statistics::Distributions::chisqrprob($df,$X2);

$p_value=1-$p_value;

my $out=$X2."\t".$p_value;

return $out;

}

######################################################################################

sub X2df1 {#

my ($A,$T)=@_;

return (abs($A-$T))**2/$T;

}

######################################################################################

sub X2df2 {#

my ($A,$T)=@_;

return (abs($A-$T)-0.5)**2/$T;

}

#######################################################################################

print STDOUT "\nDone. Total elapsed time : ",time()-$BEGIN_TIME,"s\n";

#######################################################################################

# ------------------------------------------------------------------

# sub function

# ------------------------------------------------------------------

################################################################################################################

sub ABSOLUTE_DIR{ #$pavfile=&ABSOLUTE_DIR($pavfile);

my $cur_dir=`pwd`;chomp($cur_dir);

my ($in)=@_;

my $return="";

if(-f $in){

my $dir=dirname($in);

my $file=basename($in);

chdir $dir;$dir=`pwd`;chomp $dir;

$return="$dir/$file";

}elsif(-d $in){

chdir $in;$return=`pwd`;chomp $return;

}else{

warn "Warning just for file and dir\n";

exit;

}

chdir $cur_dir;

return $return;

}

################################################################################################################

sub max{#&max(lists or arry);

#求列表中的最大值

my $max=shift;

my $temp;

while (@_) {

$temp=shift;

$max=$max>$temp?$max:$temp;

}

return $max;

}

################################################################################################################

sub min{#&min(lists or arry);

#求列表中的最小值

my $min=shift;

my $temp;

while (@_) {

$temp=shift;

$min=$min<$temp?$min:$temp;

}

return $min;

}

################################################################################################################

sub revcom(){#&revcom($ref_seq);

#获取字符串序列的反向互补序列，以字符串形式返回。ATTCCC->GGGAAT

my $seq=shift;

$seq=~tr/ATCGatcg/TAGCtagc/;

$seq=reverse $seq;

return uc $seq;

}

################################################################################################################

sub GetTime {

my ($sec, $min, $hour, $day, $mon, $year, $wday, $yday, $isdst)=localtime(time());

return sprintf("%4d-%02d-%02d %02d:%02d:%02d", $year+1900, $mon+1, $day, $hour, $min, $sec);

}

sub USAGE {#

my $usage=<<"USAGE";

ProgramName:

Version: $version

Description: X2

Usage:

Options:

-i <file> Xxx.normalized_type*.freq.txt must be given,format as " #SLAF_ID . . . dep_aa dep_ab P_aa M_aa P_ab M_ab"

-sample <str> default [P,aa,M,ab] option

-t1 <str> default [0.66:0.33] option, the proportion for "P_aa:M_aa"

-t2 <str> default [0.01:0.99] option, the proportion for "P_ab:M_ab"

-sw <int> default [3] 3:both 1:t1 2:t2 4:t1 && t2 option

-od <dir> outdir must be given

-k output filename key of outdir must be given

-h Help

USAGE

print $usage;

exit;

}
