# Supplementary material for: Development of sex-linked markers for gender identification of Actinidia arguta
Source: Sci Rep. 2023 Aug 7;13:12780. doi: 10.1038/s41598-023-39561-0 (PMC10406875; doi:10.1038/s41598-023-39561-0)
Supplement: Supplementary file 8 — Supplementary Information 8. [file 41598_2023_39561_MOESM8_ESM.pdf]

Detailed amplification results for marker aC36306 among 32 genomic DNAs from *A. arguta* plants

	Positive	Negative	Sum	Reason for negative
Male_sample	16	1	17	phenotypic male that amplifies only a female band
Female_sample	14	1	15	the band is dim, so the gender cannot be determined
Accuracy		93.75%		

Supplemental Figure

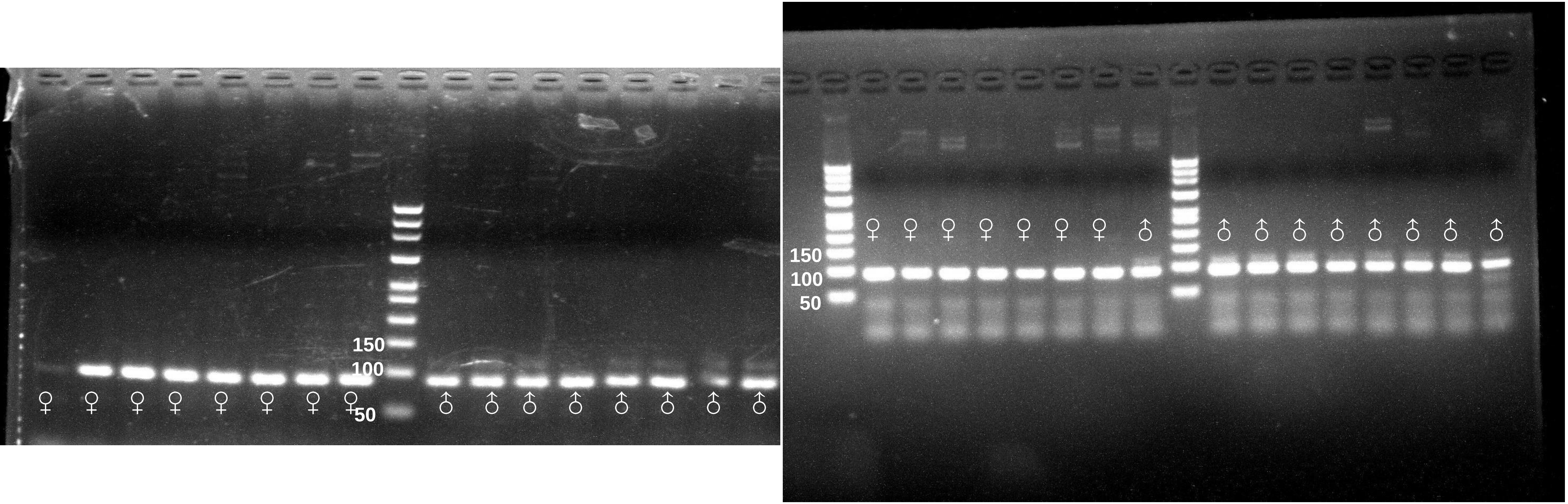

Figure S1. Amplification of 32 genomic DNAs from *A. arguta* plants by the maker aC36306. For male *A. arguta* samples, two bands are expected to be present at the gel. ♀ = female, ♂ = male.
